# Maternal immune activation induces methylation changes in schizophrenia genes

**DOI:** 10.1371/journal.pone.0278155

**Published:** 2022-11-30

**Authors:** Thomas Johnson, Defne Saatci, Lahiru Handunnetthi

**Affiliations:** 1 Wellcome Centre for Human Genetics, University of Oxford, Oxford, United Kingdom; 2 Nuffield Department of Primary Care Health Sciences, Radcliffe Observatory Quarter, University of Oxford, Oxford, United Kingdom; 3 Nuffield Department of Clinical Neurosciences, West Wing, John Radcliffe Hospital, University of Oxford, Oxford, United Kingdom; Brigham and Women’s Hospital and Harvard Medical, Channing Division of Network Medicine, UNITED STATES

## Abstract

Susceptibility to schizophrenia is mediated by genetic and environmental risk factors. Infection driven maternal immune activation (MIA) during pregnancy is a key environmental risk factor. However, little is known about how MIA during pregnancy could contribute to adult-onset schizophrenia. In this study, we investigated if maternal immune activation induces changes in methylation of genes linked to schizophrenia. We found that differentially expressed genes in schizophrenia brain were significantly enriched among MIA induced differentially methylated genes in the foetal brain in a cell-type-specific manner. Upregulated genes in layer V pyramidal neurons were enriched among hypomethylated genes at gestational day 9 (fold change = 1.57, FDR = 0.049) and gestational day 17 (fold change = 1.97, FDR = 0.0006). A linear regression analysis, which showed a decrease in gene expression with an increase in methylation in gestational day 17, supported findings from our enrichment analysis. Collectively, our results highlight a connection between MIA driven methylation changes during gestation and schizophrenia gene expression signatures in the adult brain. These findings carry important implications for early preventative strategies in schizophrenia.

## Introduction

Epidemiological studies show overwhelming evidence that both genetic and environmental risk factors are important in the aetiology of schizophrenia [1]. Recently, genome-wide association studies (GWAS) and transcriptomic studies have driven an unprecedented leap in our understanding of the disease, identifying hundreds of genetic variants associated with schizophrenia, as well as gene expression signatures in the adult schizophrenia brain [2–4]. The expression signatures in the schizophrenia brains are cell-type-specific and pathway analyses of these genes highlight the importance of neurodevelopmental processes in the aetiology of schizophrenia [3].

Epidemiological associations between maternal exposure to infections in gestation and subsequent increased risk of schizophrenia are established [5–10]. This association has been studied using maternal immune activation (MIA) models in which exposure of pregnant mice to immune insults give rise to disease-relevant pathological and behavioural changes in their adult offspring [11]. A recent study showed that MIA induction through polyI:C results in the deregulation of known schizophrenia genes in the foetal brain [12]. However, our understanding of how MIA could contribute to long lasting changes in the brain, that increase risk of schizophrenia in the offspring in adulthood, remains poorly understood.

DNA methylation regulates gene expression by recruiting proteins involved in gene repression or by inhibiting the binding of transcription factor(s) to DNA [13, 14]. Previous studies suggest that MIA during pregnancy can influence this dynamic process, providing a possible mechanism by which infection could influence gene expression in the foetal brain [15–17]. Furthermore, DNA methylation changes can persist into adulthood, resulting in long lasting modification of gene expression that may continue to influence neural function in later life [18]. This represents an attractive molecular mechanism that could link environmental exposure in early life such as maternal immune activation to adult-onset schizophrenia.

Therefore, we sought to examine the relationship between MIA-induced changes to the methylome and genes known to play a role in schizophrenia pathogenesis using published datasets. Specifically, this study investigated whether differentially expressed genes (DEGs) in schizophrenia brains and genes identified through GWAS were significantly enriched among differentially methylated genes (DMGs) in the adult offspring of pregnant mice exposed to MIA.

## Methods

### Differentially methylated genes from MIA mouse model of schizophrenia

In a previous study, DMGs were extracted from the adult offspring of pregnant mice exposed to polyI:C either in middle (POL-GD9) gestation or late (POL-GD17) gestation [17]. The prefrontal cortex (PFC) of adult mice brains were harvested for analysis at postnatal day 114 (two weeks after behavioural analysis commenced at postnatal day 100). In this study, Richetto et al. undertook logistic regression analysis of 1000 sliding base-pair regions using corrected P-values set at a threshold of <20% (q < 0.2) in the offspring of mice exposed to MIA during development. Subsequently, methylation differences at single CpG resolution were determined by considering those CpGs that were significant (q < 0.2) in the preceding analysis of the 1000 sliding base-pair regions [17]. There were 2364 and 3361 DMGs (defined as methylation change > 5%, FDR < 0.05) at POL-GD9 and POL-GD17 timepoints respectively. We included all genes with > = 1 significant differentially methylated positions. Genes that exhibited both hyper- and hypomethylation changes across different domains of the same gene were excluded (POL-GD9: 47 genes and POL-GD17: 80 genes). We found the overall trend to be that the majority of differentially methylated genes were either hypo- **or** hyper-methylated (47/2364 DMGs at POL-GD9 and 80/3361 DMGs at POL-GD17 were shown to be dually hypo- and hyper-methylated). Finally, DMGs were converted to human orthologues for downstream analyses using the BioMart database [19]. The final list of DMGs tested for enrichment is summarised in S1 File.

### Schizophrenia-associated genes from GWAS

Two sets of schizophrenia linked genes were extracted from the largest GWAS to date comprising of 76,755 patients and 243,649 controls [20]. The first set included likely causal genes (*n* = 130) that were identified through multiple fine-mapping approaches. The second set covered a broad group of credible genes (*n* = 643) with some genomic support for their role in the disease. GWAS associated genes tested for enrichment are summarised in S1 File.

### Differentially expressed genes in schizophrenia brains

Cell-type-specific gene expression patterns linked to schizophrenia were obtained from a previous single-cell RNA-sequencing study of 24 schizophrenia and 24 age- and sex-matched healthy brains [3]. The tissue samples were derived from the PFC of post-mortem brains with single cell libraries generated using the 10x Genomics Chromium Platform and sequenced using an Illumina NextSeq500 machine. Cell-type-specific gene expression patterns were available for 18 cell types, including excitatory cortical neurons, GABAergic interneurons, astrocytes, oligodendrocyte progenitor cells, microglia and endothelial cells in schizophrenia brains. Ruzicka et al. developed a new method based on the analysis of pseudo-bulk profiles to identify perturbed genes in schizophrenia. They aggregated the expression of genes characterised in each archetype-individual combination (e.g. L5/6 pyramidal neuron-schizophrenia patient) and used a linear-modelling approach to include their experimental design in the differential analysis. Single-cell pseudo-bulk analysis allowed Ruzicka et al. to compute both the mean and the variance of genes directly from single-cells. Inverse of gene variance was used as weights in their linear model. Outlier detection was also employed to exclude unreliable genes. To account for individual-specific differences, Ruzicka et al. also incorporated age, gender, PMI, batch, and medication history as covariates in their model. Finally, a threshold of FDR < 0.05 and log2(fold change) > 0.1 was used to determine whether a gene could be classified as differentially expressed. Overall, there were 1637 upregulated and 2492 downregulated genes in schizophrenia brains compared to controls across all cell types. The list of DEGs tested for enrichment are summarised in S1 File.

### Statistical analysis

We tested if schizophrenia genes (GWAS associated genes and DEGs in adult schizophrenia brains) were enriched among 1) DMGs and subsequently 2) DMGs stratified by methylation location using a hypergeometric test. The minimum overlap was set to five and the P-values were adjusted for multiple testing by controlling the false discovery rate (FDR). We further tested the correlation between DEGs and DMGs across each cell type using univariate linear regression. Q-Q plots were used to visually inspect the distribution of residuals. Normality was tested using the Shapiro-Wilk test. All analyses were carried out in R, with gene enrichment analyses conducted using the xEnricher functions in the R package ‘XGR’ (version 1.1.4).

## Results

**Fig 1:**
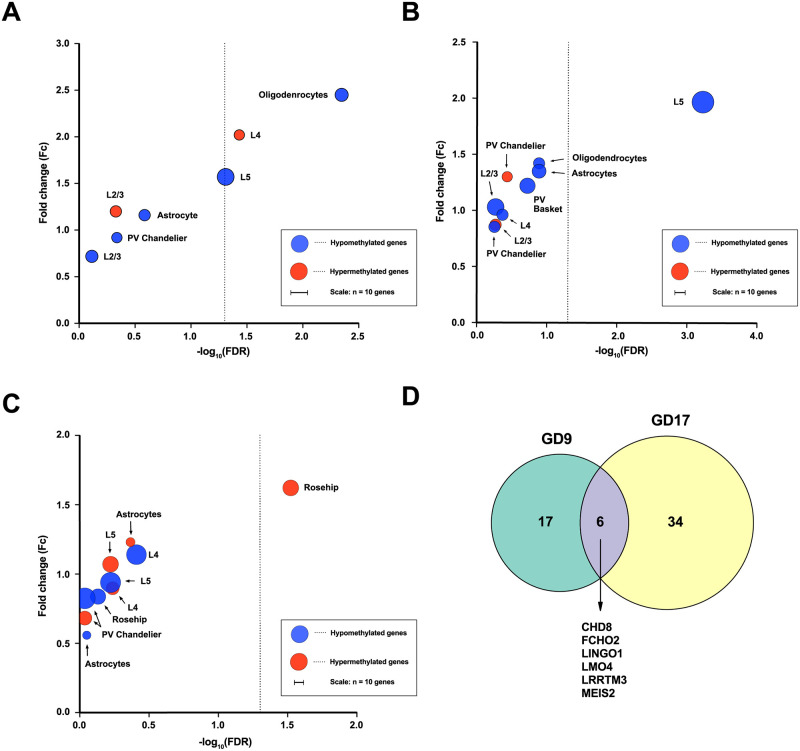
Enrichment of differentially expressed genes amongst differentially methylated foetal genes in cell subpopulations following MIA at different gestational timepoints. (A) Enrichment of upregulated genes among differentially methylated foetal genes following MIA at GD9. Size of the circles represents the number of overlapping genes. The dashed line is set at FDR = 0.05. (B) Enrichment of upregulated genes among differentially methylated foetal genes following MIA at GD17. Size of the circles represents the number of overlapping genes. The dashed line is set at FDR = 0.05. (C) Enrichment of downregulated genes among differentially methylated foetal genes following MIA at GD17. Sizes of the circles represents the number of overlapping genes. The dashed line is set at FDR = 0.05. (D) The intersect of enriched genes differentially methylated following MIA at both GD9 and GD17.

We found significant enrichment of differentially expressed schizophrenia genes among differentially methylated genes in foetal brain after MIA (Fig 1). Specifically, 1) upregulated genes in layer IV pyramidal neurons were enriched among hypermethylated genes at POL-GD9 (fold change = 2.02, FDR = 0.037), 2) upregulated genes in layer V pyramidal neurons were enriched among hypomethylated genes at POL-GD9 (fold change = 1.57, FDR = 0.049) and at POL-GD17 (fold change = 1.97, FDR = 0.0006), 3) downregulated genes expressed in GABAergic Rosehip interneurons were enriched among hypermethylated genes at POL-GD17 (fold change = 1.62, FDR = 0.03) and finally 4) upregulated genes in oligodendrocytes were enriched among hypomethylated genes at POLD-GD9 (fold change = 2.45, FDR = 0.0045). The enriched genes are summarised in S1 Table. A linear regression analysis to explore the direction of these identified associations revealed a statistically significant negative correlation between DMGs and DEGs (i.e. upregulation of genes with decrease in methylation) in layer V pyramidal neurons in POL-GD17 only (β = -0.003, p = 0.02).

When specifically looking at this enrichment when stratified by methylation sites we found that 1. Upregulated genes in layer V pyramidal neurons were enriched amongst hypomethylated ‘intron’ genes at GD9 (fold change = 1.94, FDR = 0.022) and at GD17 (fold change = 3.08, FDR = 4.9 x 10^−6^). And 2. Upregulated genes in oligodendrocytes were enriched amongst hypomethylated ‘intron’ genes at GD9 (fold change = 3.19, FDR = 0.0044) and hypomethylated ‘intergenic’ genes at GD17 (fold change = 3.27, FR = 0.004) (S3 Table).

There was no significant enrichment of GWAS associated genes among differentially methylated genes after MIA at GD9 and GD17, including fine-mapped genes (GD9-hypomethylated: fold change = 0.682, FDR = 0.79; GD17-hypomethylated: fold change = 1.41, FDR = 0.12; GD17-hypermethylated: fold change = 0.806, FDR = 0.59) or genes with broader genomic evidence (GD9-hypomethylated: fold change = 0.41, FDR = 1; GD9-hypermethylated: fold change = 0.423, FDR = 1; GD17-hypomethylated: fold change = 0.653, FDR = 1; GD17-hypermethylated: fold change = 0.479, FDR = 1).

## Discussion

In this study, we demonstrate that MIA induced methylation changes are linked to to known DEGs in schizophrenia brains. The majority of these enriched genes were specific to cortical excitatory pathways, in line with the well-established role of cortical pyramidal neurons in schizophrenia pathogenesis [21, 22]. However, we were unable to detect enrichment of GWAS-associated schizophrenia genes among the DMGs following MIA. This may be because GWAS associated genes represent a narrow group of disease relevant genes that exert their functional effects during neurodevelopment while differentially expressed genes in schizophrenia brains capture disease processes influenced by the environmental risk factors such as infections in schizophrenia pathogenesis. Overall, we show that infection induced methylation changes in the foetal brain could lead to gene expression changes in adult schizophrenia brains.

Our study shows that the effects of the interaction between MIA induced differentially methylated genes and differentially expressed schizophrenia genes are likely to be cell-type-specific. Both our enrichment and regression analyses showed that upregulated genes in layer V pyramidal neurons were associated with MIA-induced hypomethylated genes at G17. Interestingly, pathology in layer V pyramidal neurons, such as reduced dendritic field size, have previously been associated with schizophrenia pathogenesis [23]. In addition, subunit composition of glutamate receptors has shown to be altered in layer V pyramidal neurons of the prefrontal cortex in schizophrenia patients [24]. Interestingly, transcriptome profiling this layer in schizophrenia patients revealed unique abnormalities in pathways involved in stress-related regulation of translation initiation and could help to explain how stressful stimuli such as MIA contribute to pathological changes in the developing schizophrenia brain [25].

The enriched genes we identified in cortical layer V pyramidal neurons play key roles in biological processes that are relevant to schizophrenia such as neurodevelopment, synaptic connectivity, and mitochondrial function (summarised in S2 Table) [26–28]. For example, we found TCF4, encoding transcription factor 4, to be hypomethylated at GD9 and enriched amongst upregulated genes expressed by layer V pyramidal neurons. Large GWAS studies have repeatedly identified several non-coding SNPs in the 5’ located introns of the TCF4 gene that contribute to an increased risk of schizophrenia [29–31]. Furthermore, an analysis of the genomic locations of TCF4 binding sites showed significant overlap with known genetic risk factors for schizophrenia [32]. Consistent with our results, TCF4 transcript levels have been shown to be moderately increased in schizophrenia brains, suggesting long term upregulation of TCF4 expression [33, 34]. Overall, methylation changes to gene regulators such as TCF4 during neurodevelopment suggests that MIA could precipitate a network of abnormal gene-expression changes beyond those linked directly to the disease through GWAS.

Interestingly, our results suggest that differentially methylated genes in the gene body, specifically introns, are influenced by MIA. Differentially methylated intronic sites have previously been implicated in schizophrenia pathogenesis [35]. It has been demonstrated that differentially methylated regions in intronic sites have enhancer-like chromatin features in the brain [35]. Thus, a potential mechanism by which differentially methylated regions in intronic sites regulate cell-type-specific gene expression is by having these enhancer-like properties [35]. Furthermore, DNA methylation of intronic sites has been associated with increased gene expression in dividing cells, which may be particularly pertinent in the context of the influence of MIA on the developing brain [36, 37].

Additionally, our results show that hypermethylated genes at GD9 were significantly enriched amongst upregulated genes in cortical layer IV neurons. First, this result should be interpreted with caution given our regression analysis did not support this enrichment and also because the FDR of the enrichment analysis was close to the significance threshold of 0.05. However, it is also important to highlight that there is increasing evidence for the role of hypermethylation in upregulation of gene expression. For example, a comparison of genome-wide methylation and gene expression in different tissues demonstrated that a significant minority of hypermethylated regions correlated with increased expression of associated genes [38].

There were several limitations in this study. Our analysis was limited by the quality of the available data, including DMGs from the MIA models, the GWAS-associated genes, and DEGs extracted from the single-cell RNA sequencing study. Further, there are clear translational differences between mice and humans which impedes the ability of polyI:C to fully recapitulate the complexity of immune responses during pregnancy. Finally, we only investigated MIA-induced gene expression changes in one model of MIA mimicking viral infection. Therefore, further work is needed to understand how other models of MIA involving various immune agents could affect the methylation of schizophrenia genes.

In summary, this study provides novel insights into the epigenetic mechanisms underpinning the interplay between genetic and environmental risk factors in the aetiology of schizophrenia. Our results suggest that MIA can induce stable DNA methylation changes that could lead to cell-type-specific gene expression changes in schizophrenia brains. These genes are linked to abnormal neurodevelopment, impaired synaptic connectivity, and mitochondrial dysfunction. Importantly, these findings carry clear implications for disease prevention strategies in schizophrenia because treatment and/or prevention of maternal infection during pregnancy are tractable health goals.

## Supporting information

S1 File Summary of differentially expressed genes and GWAS associated genes tested for enrichment amongst differentially methylated genes at GD9 and GD17. (XLSX)

S1 Table Summary of differentially expressed genes from each cell type enriched amongst differentially methylated genes identified at GD9 and GD17. (DOCX)

S2 Table Descriptive summary of enriched differentially expressed genes. (DOCX)

S3 Table Summary of enrichment analysis results of differentially expressed genes from each cell type amongst all differentially methylated genes and differentially methylated gene stratified by CpG site identified at GD9 and GD17. (DOCX)
