# Supplementary material for: Maternal immune activation induces methylation changes in schizophrenia genes
Source: PLoS One. 2022 Nov 30;17(11):e0278155. doi: 10.1371/journal.pone.0278155 (PMC9710780; doi:10.1371/journal.pone.0278155)
Supplement: S3 Table — (DOCX) [file pone.0278155.s004.docx]

*Supplementary Table 3*

|  | | **All CpG Sites** | | **Intron** | | **Exon** | | **Intergenic** | |
| --- | --- | --- | --- | --- | --- | --- | --- | --- | --- |
| **GD9** | | | | | | | | | |
| **Cell Type** | **+/-** | **Hyper-** | **Hypo** | **Hyper-** | **Hypo-** | **Hyper-** | **Hypo-** | **Hyper-** | **Hypo-** |
| **All upregulated** |  |  |  |  |  |  |  |  |  |
| **All downregulated** |  |  |  |  |  |  |  |  |  |
| **Excitatory cells** |  |  |  |  |  |  |  |  |  |
| **Inhibitory cells** |  |  |  |  |  |  |  |  |  |
| **Ex-NRGN** |  |  |  |  |  |  |  |  |  |
| **Ex-SZTR** |  |  |  |  |  |  |  |  |  |
| **L23** | Up |  |  |  |  |  |  |  |  |
|  | Down |  |  |  | Overlap = 27, Fc = 1.82, FDR = 0.0018 |  |  |  |  |
| **L4** | Up | Overlap = 5, Fc = 2.02, FDR = 0.037 |  |  |  |  |  |  |  |
|  | Down |  |  |  | Overlap = 18, Fc = 1.98, FDR = 0.0019 |  |  |  |  |
| **L5** | Up |  | Overlap = 13, Fc = 1.51, FDR = 0.049 |  | Overlap = 8, Fc = 1.94, FDR = 0.022 |  |  |  |  |
|  | Down |  |  |  |  |  |  |  |  |
| **L56** |  |  |  |  |  |  |  |  |  |
| **L56CCa** | Up |  |  |  |  |  |  |  |  |
|  | Down |  |  |  | Overlap = 24, Fc = 1.55, FDR = 0.011 |  |  |  |  |
| **L56CCb** |  |  |  |  |  |  |  |  |  |
| **In-Rosehip** |  |  |  |  |  |  |  |  |  |
| **In-VIP** | Up |  |  |  |  |  |  |  |  |
|  | Down |  |  |  | Overlap = 6, Fc =  2.17, FDR =  0.02 |  |  |  |  |
| **In-Reelin** | Up |  |  |  |  |  |  |  |  |
|  | Down |  |  |  | Overlap = 11, Fc = 1.64, FDR = 0.037 |  |  |  |  |
| **In-PV (Basket)** | Up |  |  |  |  |  |  |  |  |
|  | Down |  |  |  | Overlap = 22, Fc = 1.49, FDR = 0.022 |  |  |  |  |
| **In-PV (Chandelier)** |  |  |  |  |  |  |  |  |  |
| **In-SST** | Up |  |  |  |  |  |  |  |  |
|  | Down |  |  |  | Overlap = 15, Fc = 1.98, FDR = 0.0039 |  |  |  |  |
| **Ast** |  |  |  |  |  |  |  |  |  |
| **OPC** |  |  |  |  |  |  |  |  |  |
| **Oli** | Up |  | Overlap = 8, Fc = 2.45, FDR = 0.0045 |  | Overlap = 5, Fc = 3.19, FDR = 0.004 |  |  |  |  |
|  | Down |  |  |  |  |  |  |  |  |
| **Mic** |  |  |  |  |  |  |  |  |  |
| **Endo** |  |  |  |  |  |  |  |  |  |

| **GD17** | | | | | | | | | |
| --- | --- | --- | --- | --- | --- | --- | --- | --- | --- |
| **Cell Type** | **+/-** | **Hyper-** | **Hypo-** | **Hyper-** | **Hypo-** | **Hyper-** | **Hypo-** | **Hyper-** | **Hypo-** |
| **All upregulated** |  |  |  |  | Overlap = 56, Fc = 1.49, FDR = 0.0015 |  |  |  |  |
| **All downregulated** |  |  |  |  |  |  |  |  |  |
| **Excitatory cells** | Up |  |  |  | Overlap = 32, Fc = 1.43, FDR = 0.03 |  |  |  |  |
|  | Down |  |  |  |  |  |  |  |  |
| **Inhibitory cells** | Up |  |  |  | Overlap = 18, Fc = 1.61, FDR = 0.032 |  |  |  |  |
|  | Down |  |  |  |  |  |  |  |  |
| **Ex-NRGN** |  |  |  |  |  |  |  |  |  |
| **Ex-SZTR** |  |  |  |  |  |  |  |  |  |
| **L23** |  |  |  |  |  |  |  |  |  |
| **L4** |  |  |  |  |  |  |  |  |  |
| **L5** | Up |  | Overlap = 22, Fc = 1.97, FDR = 6 x 10^-4^ |  | Overlap = 18, Fc = 3.08, FDR = 4.9 x 10^-6^ |  |  |  |  |
|  | Down |  |  |  |  |  |  |  |  |
| **L56** | Up |  |  |  | Overlap = 7, Fc = 1.95, FDR = 0.027 |  |  |  | Overlap = 5, Fc = 2.01, FDR = 0.037 |
|  | Down |  |  |  |  |  |  |  |  |
| **L56CCa** |  |  |  |  |  |  |  |  |  |
| **L56CCb** | Up |  |  |  | Overlap = 9, Fc = 1.99, FDR = 0.014 |  |  |  |  |
|  | Down |  |  |  |  |  |  |  |  |
| **In-Rosehip** | Up |  |  |  |  |  |  |  |  |
|  | Down | Overlap = 18, Fc = 1.62, FDR = 0.03 |  |  |  |  |  |  |  |
| **In-VIP** |  |  |  |  |  |  |  |  |  |
| **In-Reelin** |  |  |  |  |  |  |  |  |  |
| **In-PV (Basket)** | Up |  |  |  |  |  |  |  |  |
|  | Down |  |  |  | Overlap = 30, Fc = 1.44, FDR = 0.034 |  |  |  |  |
| **In-PV (Chandelier)** | Up |  |  |  |  |  |  |  |  |
|  | Down |  |  |  |  |  | Overlap = 5, Fc = 2.16, FDR = 0.027 |  |  |
| **In-SST** | Up |  |  |  |  |  |  |  |  |
|  | Down |  |  |  | Overlap = 17, Fc = 1.58, FDR = 0.044 |  |  |  |  |
| **Ast** | Up |  |  |  | Overlap = 7, Fc = 2, FDR = 0.023 |  |  |  |  |
|  | Down |  |  |  |  |  |  |  |  |
| **OPC** |  |  |  |  |  |  |  |  |  |
| **Oli** | Up |  |  |  |  |  |  |  | Overlap = 5, Fc = 3.27, FDR = 0.004 |
|  | Down |  |  |  |  |  |  |  |  |
| **Mic** |  |  |  |  |  |  |  |  |  |
| **Endo** | Up |  |  |  | Overlap = 5, Fc = 2.64, FDR = 0.011 |  |  |  |  |
|  | Down |  |  |  |  |  |  |  |  |
